# Cost-comparison analysis of diffusion weighted magnetic resonance imaging (DWMRI) versus second look surgery for the detection of residual and recurrent cholesteatoma

**DOI:** 10.1186/s40463-019-0384-1

**Published:** 2019-11-07

**Authors:** David L. Choi, Michael K. Gupta, Ryan Rebello, Jason D. Archibald

**Affiliations:** 1 Division of Otolaryngology – Head and Neck Surgery, Department of Surgery, McMaster University, Hamilton, Ontario Canada; 2 Department of Radiology, McMaster University, Hamilton, Ontario Canada

**Keywords:** Cholesteatoma, Recurrence, Diffusion-weighted MRI, Second-look surgery

## Abstract

**Background:**

Cholesteatoma is a destructive, erosive growth of keratinizing squamous epithelium in the middle ear cleft. Following treatment with a canal wall-up (CWU) tympanomastoidectomy, surveillance of residual and recurrent disease has traditionally been achieved through a second look tympanotomy following the initial procedure. Historically, MRI sequences have been inadequate at differentiating between granulation tissue, inflammation, and cholesteatoma. Recent literature has shown diffusion-weighted magnetic resonance imaging (DWMRI) to be a viable alternative to second look surgery for the detection of residual or recurrent disease. The goal of the present study was to perform a cost analysis of DWIMRI versus second look surgery in the detection of residual or recurrent cholesteatoma following combined approach tympanomastoidectomy.

**Methods:**

A probabilistic decision tree model was generated from a literature review to compare traditional second look surgery with DWMRI. Cost inputs were obtained from the Ontario Case Costing Initiative, the Ontario Health Insurance Plan (OHIP) schedule of benefits. Costs were reported in Canadian dollars and a payer perspective was adopted. A probabilistic sensitivity analysis was performed.

**Results:**

According to the probabilistic sensitivity analysis, mean cost difference of traditional second look tympanotomy versus echo planar imaging (EPI) DWMRI was $180.27CAD, 95%CI [$177.32, $188,32] in favour of second-look tympanotomy. However, mean cost difference of traditional second look tympanotomy versus non-EPI DWMRI was $390.66CAD, 95%CI [$381.52, $399.80] in favour of non-EPI DWMRI.

**Conclusions:**

Diffusion-weighted MRI, specifically non-EPI sequences, are a viable cost-saving alternative to second-look tympanotomy in the setting of detecting residual or recurrent cholesteatoma.

## Introduction

Cholesteatoma is a destructive, erosive growth of keratinizing squamous epithelium in the temporal bone. Disease may commonly involve the middle ear space, but can also present in any anatomical location of the temporal bone including the external auditory canal, mastoid antrum, and petrous apex [1]. Growth and expansion of a cholesteatoma may result in infection, otorrhea, ossicular and bone destruction, hearing loss, facial nerve paralysis, labyrinth fistula, and intracranial complications. This destructive potential of cholesteatoma is mediated through two predominant pathophysiologic mechanisms. First, epidermal debris can cause pressure-induced bone resorption [2]. Second, cytokine-mediate inflammation can cause enzymatic dissolution of bone [1, 3].

The first and fundamental principle of cholesteatoma management is the surgical removal of disease to prevent further extension of disease. At the same time, complications of cholesteatoma can be addressed while preserving normal anatomy. After these two components have been dealt with, preservation or improvement of hearing can then be addressed. Selection of surgical procedure is consequently dependent on the location and extent of disease [1]. Small and limited cholesteatomas without complications can be treated with atticotomy and tympanoplasty [1, 4]. For more extensive disease, a tympanomastoidectomy may be the treatment of choice for complete disease removal. Typically, surgeons will elect to perform a combine approach or “canal wall-up” (CWU) tympanomastoidectomy in which the posterior wall of the external auditory canal is preserved. In cases of extensive erosive disease that dictate improved exposure for disease removal, a “canal wall-down” (CWD) mastoidectomy is necessary. This will create a mastoid cavity potentially requiring lifelong maintenance by an otolaryngologist. Given higher rates of recurrence, disease surveillance after CWU tympanopastoidectomy is accomplished through a surgical re-exploration and direct visualization of the disease site [2, 5, 6]. This is often known as a “second look tympanotomy” procedure, and often will take place 6–18 months following the primary surgical extirpation of disease. This second procedure will often also incorporate an ossicular chain reconstruction to re-establish or improve hearing [7].

**Fig. 1:**
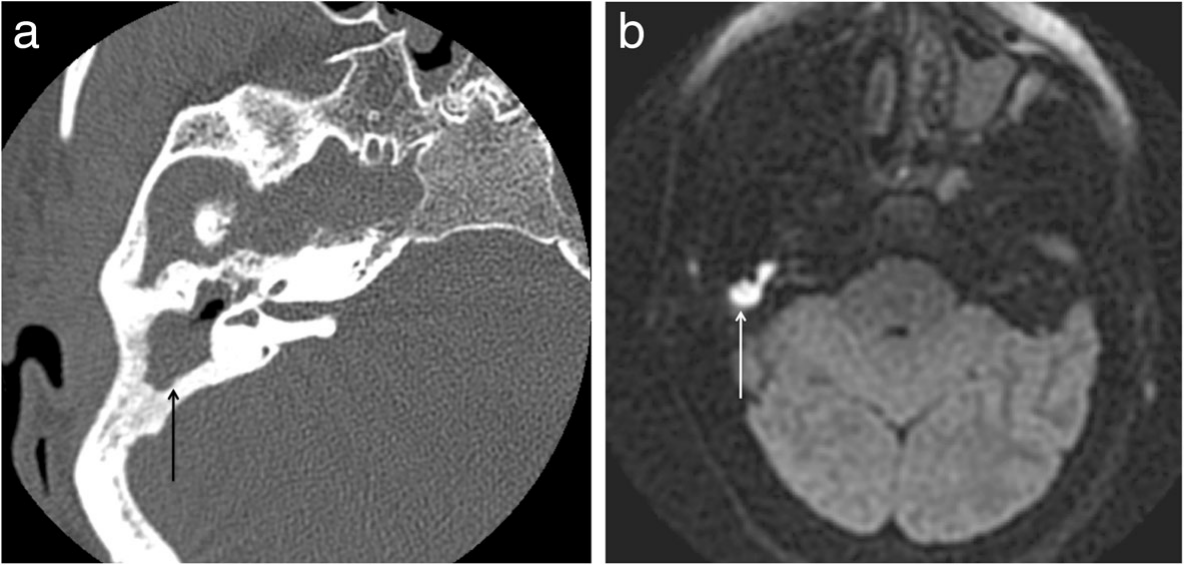
Patient with recurrent cholesteatoma following a canal-wall up tympanomastoidectomy. A) Axial unenhanced small field of view CT image demonstrates low density soft tissue (black arrow) filling the attic, aditus ad antrum and epitympanum. B) Axial 3 mm thick PROPELLER non-EPI DWMRI shows avid restricted diffusion (white arrow) within the recurrent cholesteatoma in the attic and epitympanum

The utility of traditional magnetic resonance imaging (MRI) in the setting of cholesteatoma have been hampered by poor specificity. Disadvantages include lacking the capability of differentiating between true disease and post-surgical granulation tissue, fibrosis, or fluid. However, recent advances in MR imaging have greatly improved the ability to detect recurrent and residual cholesteatoma. Diffusion-weighted MRI (DWMRI) is a form of imaging built on the concept of random Brownian motion of water molecules within tissue. Tissues through which water molecules can easily diffuse, such as cerebrospinal fluid, will have no significant net motion in any direction and will produce no signal and will appear hypointense. In contrast, tissues where water molecules have difficulty diffusing, such as cholesteatoma, will appear bright (Fig. 1). This phenomenon seen in cholesteatomas is known as restriction of diffusion. The advent of diffusion-weighted MRI (DWMRI), particularly with non-echo planar (non-EPI) protocol, has been suggested as a viable alternative to second look surgery given its superb sensitivity and specificity [8–10]. This diagnostic accuracy and ability to differentiate between post-surgical granulation and recidivism has led to its increased use in the otology community for follow-up after initial surgical treatment due to its non-invasive nature in comparison to second-look exploration.

Non-EPI DWMRI represents an alternative to second look surgery, and may be a cost-saving alternative for monitoring of residual and recurrent cholesteatomas. The objective of this study is to compare local costs of DWMRI versus traditional second look surgery in a Canadian tertiary care institution using a cost-analysis probability tree model. To the authors’ knowledge, this is the first cost analysis comparing DWMRI to surgery in the detection of residual and/or recurrent cholesteatoma.

## Methods

A comprehensive search of the literature was performed through the electronic databases Medline (1945–2015), EMBASE (1948–2015), Cochrane Central Register of Controlled Trials and Google Scholar. Medical subject headings “cholesteatoma,” “tympanomastoidectomy,” “revision,” “recurrence,” “second look surgery,” “MRI,” and “tympanotomy” were utilized in various combinations. Individual sensitivity, specificity, positive predictive value, and negative predictive values were extracted, combined, and utilized for a probabilistic cost analysis model. This study was exempt from institutional ethics board review given that no individual personal patient data was utilized at any point.

Aggregate annual mean case cost data was obtained from provincial case costing initiative services, the institutional case costing department, the hospital department of diagnostic radiology, and Ontario Health Insurance Program (OHIP) Schedule of Benefits for the year 2016. Cost data was obtained for DWMRI, second look tympanotomy and revision tympanomastoidectomy. For DWMRI, the institutional case costing department provided average materials and cost-per-scan totals for patients who underwent DWMRI in the 2016 fiscal year. This was then added to the radiologist OHIP billing fees. For operative case data, average materials, operating room costs, perioperative costs, and anesthesia costs were added to OHIP billing fees for surgeons to generate fee totals (see Results).

**Fig. 2:**
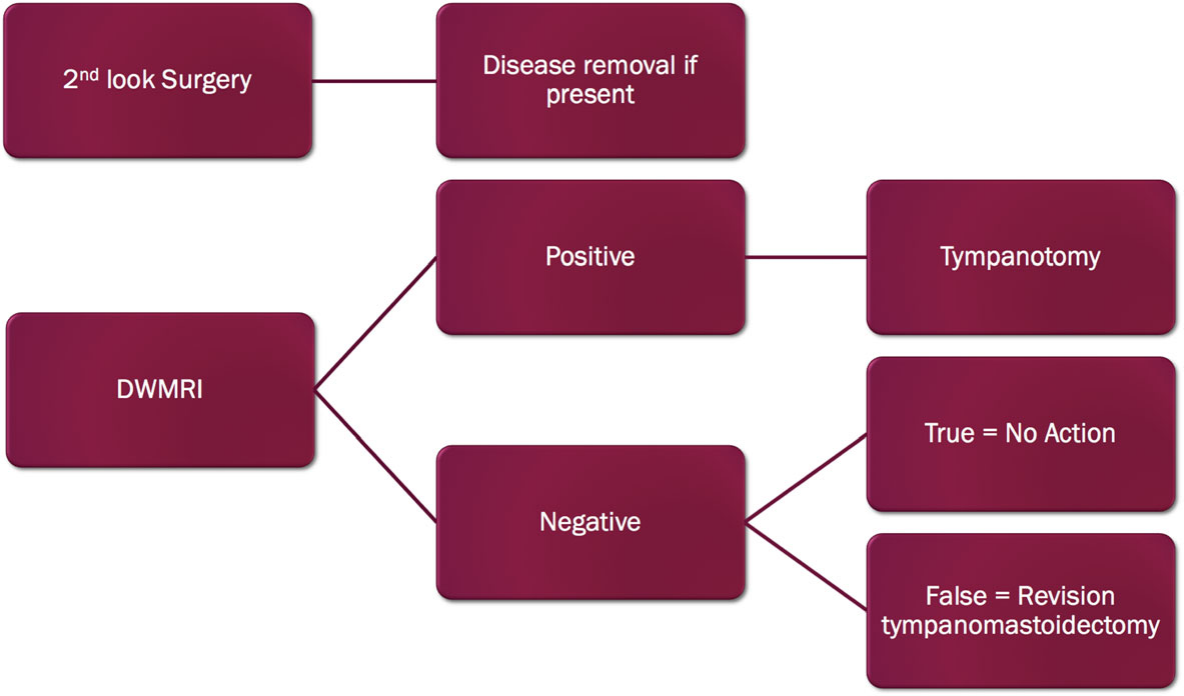
Cost-analysis decision tree model used in probabilistic sensitivity analysis

A hypothetical decision-tree model was generated by the authors (Fig. 2). This model highlights main decision treatments based on mode of follow-up. Due to its hypothetical nature, the nature of this study makes several assumptions. First, both modes of follow-up occur within the first year following the initial surgery; disease load is often small and non-extensive. Therefore, patients who undergo second look tympanostomy as the initial mode of disease surveillance will have small-volume disease removed if present and typically not require tympanomastoidectomy for extirpation of extensive disease. Second, patients who are monitored by DWMRI that are falsely negative will likely require revision tympanomastoidectomy as the disease will likely become more extensive. It is imperative to stress that these assumptions were mandatory in creating of this hypothetical decision tree as an infinite number of combinations of surgeries and subsequent follow-up DWMRIs are possible. This probabilistic model assumes best clinical practice and patient adherence based on these assumptions to allow for the ability to calculate cost.

A probabilistic cost analysis on Microsoft Excel (Microsoft Corp., 2016) was performed. Variables relating to predictive value of the tests were varied according to standard error calculated from the results of the literature review. Predictive values were varied on a beta-inverse distribution. Costs were varied on a gamma distribution. Standard error estimated to be an assumed 25% of the mean for the majority of cost components in our model. Non-anesthetic cost data retrieved from OHIP Schedule of benefits were not varied. The probabilistic model was run five thousand times to generate costing data with 95% confidence intervals.

## Results

A total of 16 articles addressing test characteristics of DWMRI for detection of residual and recurrent cholesteatoma were identified after abstract screening. One article was excluded as it did not include individualized data regarding test characteristics. Fifteen studies were included into the final probabilistic analysis (Table 1). Seven studies used EPI sequence imaging while eight studies utilized non-EPI sequence imaging. A total of 221, and 122 individual diagnostic tests were collected for EPI and non-EPI sequences respectively. Aggregate values for sensitivity, specificity, positive predictive value, and negative predictive value were calculated from the results of the literature review. Values were individually calculated for EPI sequences and non-EPI sequences (Table 2). Mean costs of second look tympanotomy, revision tympanomastoidectomy, and DWMRI were estimated to be $2695.28, $3964.81, and $686.10, respectively.

**Table 1:** Studies from Literature Review and Diagnostic Properties

Study	DWMRI Type (EPI vs. non-EPI)	Patients included (n)	Sensitivity	Specificity
Pizzini et al., 2010 [11]	Non-EPI	11	1.00	1.00
Huins et al., 2010 [12]	Non-EPI	18	0.86	1.00
Rajan et al., 2010 [13]	Non-EPI	15	1.00	1.00
Plouin-Gaudon et al., 2010 [14]	Non-EPI	21	0.62	0.88
Lehmann et al., 2009 [15]	Non-EPI	10	1.00	0.50
Dhepnorrarat et al., 2009 [16]	Non-EPI	23	1.00	1.00
Dubrulle et al., 2006 [10]	Non-EPI	24	1.00	0.91
Cimsit et al., 2010 [17]	EPI	26	1.00	0.93
Jindal et al., 2010 [18]	EPI	35	0.83	0.82
Venail et al., 2008 [19]	EPI	31	0.60	0.73
Toyama et al., 2015 [20]	EPI	17	0.92	0.60
Jeunen et al., 2008 [21]	EPI	32	0.55	0.90
Vercruysse et al., 2006 [22]	EPI	45	0.13	1.00
Stasolla et al., 2004 [23]	EPI	18	0.86	1.00
Aikele et al., 2003 [24]	EPI	17	0.77	1.00

**Table 2:** Results of aggregate diagnostic test characteristics for DWMRI from literature review

Imaging sequence	True positives	False positives	False negatives	True negatives	Sensitivity	Specificity	Positive predictive value	Negative predictive value
EPI sequences (*n* = 221)	78	10	33	100	0.70	0.91	0.89	0.75
Non-EPI sequences (*n* = 122)	56	3	7	56	0.89	0.95	0.95	0.89

After running a probabilistic model five thousand times (Fig. 2), the aggregate mean cost of the EPI imaging arm versus traditional second look surgery arm was $2871.89CAD versus $2691.62CAD. This was a cost difference of $180.27CAD in favour of the second look surgery arm (Table 3). The aggregate mean cost of non-EPI imaging versus traditional second look surgery was $2298.21CAD versus $2688.87CAD. This is a cost difference of $390.66CAD in favour of non-EPI DWMRI (Table 4).

**Table 3:** Costs from probabilistic analysis of EPI sequence DWMRI

	EPI DWMRI Costs (CAD)	Surgery Costs (CAD)	Cost difference (CAD)
Mean (Standard deviation)	2871.89 (*s* = 342.27)	2691.62 (*s* = 490.82)	+ 180.27
95% CI	[2861.04, 2882.75]	[2676.06, 2707.8]	[+ 172.23, + 188.32]

**Table 4:** Costs from probabilistic analysis of non-EPI sequence DWMRI

	Non-EPI DWMRI Costs (CAD)	Surgery Costs (CAD)	Cost difference (CAD)
Mean (Standard deviation)	2298.21 (*s* = 292.91)	2688.87 (*s* = 487.71)	−390.66
95% CI	[2288.92, 2307.50]	[2673.40, 2704.33]	[−381.52, − 399.80]

## Discussion

The cornerstone of cholesteatoma management is surgical removal of disease with the primary goal of producing a safe and disease-free ear. Secondary goals of surgery are to preserve or restore hearing [25]. Although CWD procedures offer unparalleled access to the middle ear for disease removal, they are often accompanied by significant disadvantages [26]. These include delayed wound healing, chronic otorrhea, inadequate contouring of the external ear for hearing amplification devices, and potentially poorer outcomes compared to patients with CWU tympanomastoidectomies due to differences in acoustic transmission [27]. In order to mitigate and reduce these complications, otologists often will elect to perform CWU surgery with the knowledge that there are increased rates of residual and recurrent disease. Published literature has examined the rates of recurrence and residual disease in CWD versus CWU procedures. Early literature has shown high rates of recidivism, with up to 12.4% of CWD and 42% of CWU procedures resulting recurrence or residual disease [28]. In contrast, newer studies within the last two decades have shown an overall decrease in recurrence rates, with CWD mastoidectomy recurrence rates as low as 7% [29]. In a meta-analysis of 4720 patients, Tomlin et al. has shown that CWD procedures have a 2.87 times relative risk of recidivism compared to CWU procedures and advocated for surgical exploration in second-look procedures [5].

Following CWU tympanomastoidectomy for cholesteatoma, the otologic surgeon is typically faced with three possible scenarios with varying postoperative management strategies. Within the first group, the surgeon has clearly left residual cholesteatoma, of which revision surgery is certainly required. In the second group, the surgeon has completely and confidently removed all trace of disease and clinical surveillance and monitoring will suffice. The third group, in which the extent of disease removal is indeterminant, presents a management dilemma. Recurrent and residual cholesteatoma is a considerable risk to the patient if left undetected and subsequently not managed. Within this third group of patients, surveillance has classically been through a second look surgery, comprising of a tympanotomy and potential removal of recurrent or residual disease if it is present.

Interest within the last several decades has focused on advanced imaging techniques in order to accurately detect cholesteatoma in the post-operative setting to avoid complications and morbidity associated with surgery. With the advent of advanced imaging techniques, non-invasive methods have been introduced into the diagnostic algorithm for the detection of disease after initial cholesteatoma removal. Delayed postcontrast T1-weighted MR imaging was one of the first imaging techniques used to detect cholesteatoma, relying on the lack of contrast enhancement in cholesteatomas. The lack of late enhancement ruled out fibrotic inflammatory granulation tissue and scarring [30]. Subsequent development of EPI DWMRI, which is commonly utilized for intracranial imaging, was suggested for cholesteatoma use. However, susceptibility artifacts at multiple air-bone interfaces in the skull base and low spatial resolution severely hamper sensitivity when lesions are smaller than 5 mm [22].

Non-EPI DWMRI within the last decade has largely supplanted EPI in the detection of postoperative cholesteatoma due to higher inherent resolution, thinner slice thickness, and fewer susceptibility artifacts. These properties all contribute to the improved accuracy in cholesteatoma detection with sensitivities of 90–100% for cholesteatomas as small as 2 mm [31]. Specifically, a systematic review by Jindal et al. demonstrated that non-EPI DWMRI has a sensitivity, specificity, positive predictive, and negative predictive value of 91.4, 95.8, 97.3, and 85.2% respectively [32]. In this current study, this improved diagnostic ability of non-EPI imaging to detect disease is responsible for over $600 in cost-savings in comparison to EPI imaging. Whereas EPI imaging represents a costlier alternative to surgery due to its lower diagnostic ability, non-EPI represents nearly $400 in cost-savings within our probabilistic model. This is in support for using non-EPI DWMRI in the correct patient and avoids the potential risks and complications of revision ear surgery, such as facial nerve injury, hearing loss, infection, bleeding, and requirement of a general anesthetic.

Cholesteatoma represents a common disease entity for otolaryngologists and subspecialty otologists, and interest has been generated around the cost of care for patients with this pathology. A retrospective review from 2013 in the United States estimated that the average hospital charge per patient per year was $10000USD [33]. Studies have also examined the use of different surgical approaches on cost. Bennett et al. reviewed Medicare and Medicaid reimbursements for intraoperative endoscopic surveillance of cholesteatoma and noted endoscopic surveillance ($6100USD) to be less expensive than traditional second look surgeries ($11,829.83USD) and annual MRI ($9891.95USD) per patient [34]. This echoes the findings in the current study that DWMRI offers cost savings compared to surgery. Unfortunately, endoscopic surveillance is not currently performed routinely as a primary modality of surveillance within our institution to generate costing data. Furthermore, no separate billing codes exist for a purely endoscopic approach to disease surveillance at this time. Future cost-analysis studies would benefit from examining an endoscopic surveillance versus DWMRI.

This study is not without its limitations. Certainly, a wide multitude of clinical outcomes are possible with a false negative DWMRI, especially if not detected in clinical follow-up in the future. However, the possibility of this causing catastrophic consequences in the setting of a clinically astute clinician is exceedingly low. We therefore considered these costs to be negligible in order to perform our probabilistic analysis. As previously mentioned, a probabilistic model cannot factor in all possible clinical outcomes, and rather incorporates a reasonable best-practice pathway to estimate costs in a sensitivity analysis. Therefore, after careful consideration, it was the intent of the authors to purposefully only incorporate the initial follow-up of disease with either DWMRI or surgery. We recognize the possibility of multiple follow-up scans and surgeries over time, but an assumption was made that a single comparison of cost in one instance can be inferred to subsequent decisions between diagnostic modalities, given similar probabilistic conditions. In our analysis, we also did not address the need for second surgery in the setting of prior ossicular chain erosion removal necessitating the need for a subsequent ossiculoplasty, regardless of the presence of cholesteatoma. For these patients, the need for DWMRI may be unnecessary, and the cost of either diagnostic modality would not be a factor in consideration.

A growing number of practicing cholesteatoma surgeons advocate for non-classical surgical approaches such as CWD surgery in combination with canal wall reconstruction and/or mastoid obliteration. A systematic review of the literature of 146 studies with 1534 patients examined rates of residual and recurrent cholesteatoma after single-stage CWU and CWD with mastoid obliteration found acceptable rates of recurrence of approximately 4–6% [35]. Roux et al. examined rates of residual and recurrent disease in a consecutive cohort of 36 ears and found the recurrence and residual rates of cholesteatoma after a mean follow-up of 24 months to be 3.1% and 6.2, respectively [36]. As the current study focus was to differentiate costing options between DWMRI and second look, we did not differentiate between such patients who underwent differing initial cholesteatoma surgeries in our probabilistic model. Future prospective or retrospective patient costing studies could examine the difference between these and more classical surgical methods, and whether individual patient-related costs are different within a subgroup analysis.

Moreover, surgical recovery and opportunistic costs for the patient have not been factored in due study design and methodologic difficulties in assigning cost to patient values. Future studies could examine and compare the cost-effectiveness or cost-utility of each of these arms in the decision model and consider timing as a factor.

## Conclusion

Although various authors have now reported on the efficacy and diagnostic characteristics of DWMRI and specifically non-EPI sequences for cholesteatoma surveillance, this is the first cost-savings analysis comparing and demonstrating a benefit for non-EPI DWMRI over traditional surgical approaches [7, 10, 28]. As gatekeepers of health care resources, it is imperative for otolaryngologists, radiologists, and primary care physicians to be aware of the role of non-EPI as well as its potential cost benefit over a surgical exploration of the disease site.

Our study results become quite relevant in a universal healthcare model, such as that which exists in Canada. In this setting, operative resources are often limited and wait times to surgery can be significant. Effective alternatives to surgery can be extremely advantageous in disease management. Non-EPI DWMRI may infer cost-savings to health care systems and potentially avoid utilization of costly operative resources.

## Data Availability

The datasets used and/or analysed during the current study are available from the corresponding author on reasonable request.
